# Supplementary material for: Laparogastroscopy—A Transgastric Laparoscopic Approach for Malignant Esophageal Stenosis
Source: Healthcare (Basel). 2023 Mar 9;11(6):815. doi: 10.3390/healthcare11060815 (PMC10048741; doi:10.3390/healthcare11060815)
Supplement: Supplementary file 1 [file healthcare-11-00815-s001.zip › healthcare-2177265-supplementary.pdf]

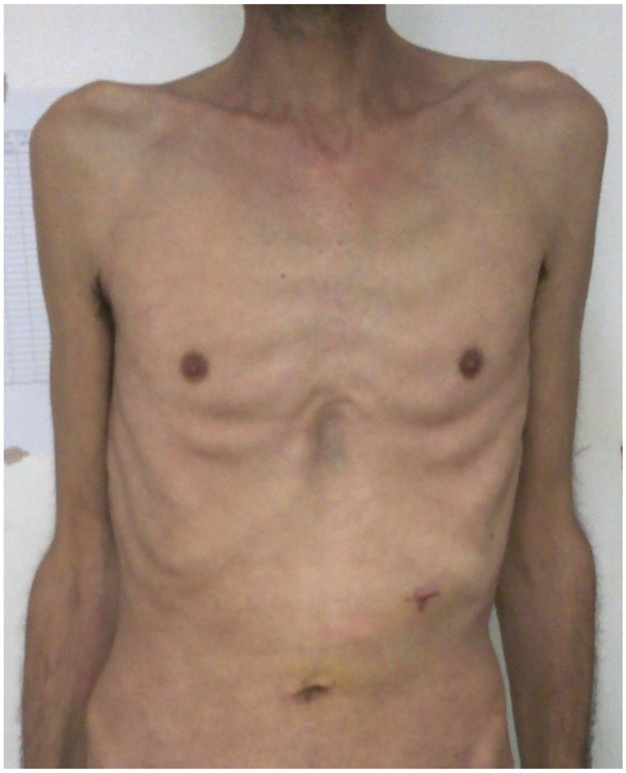

**Figure S1**. Cachectic patient postoperative image.
Patient with malignant esophageal stenosis, inoperable oncologically, possibly unresectable, with or without fistula to the trachea, avoided by gastroenterologists for topographic reasons, for stenosis stage reasons or even for financial reasons (sometimes very high price of SEMS), was stented by laparogastroscopic method.

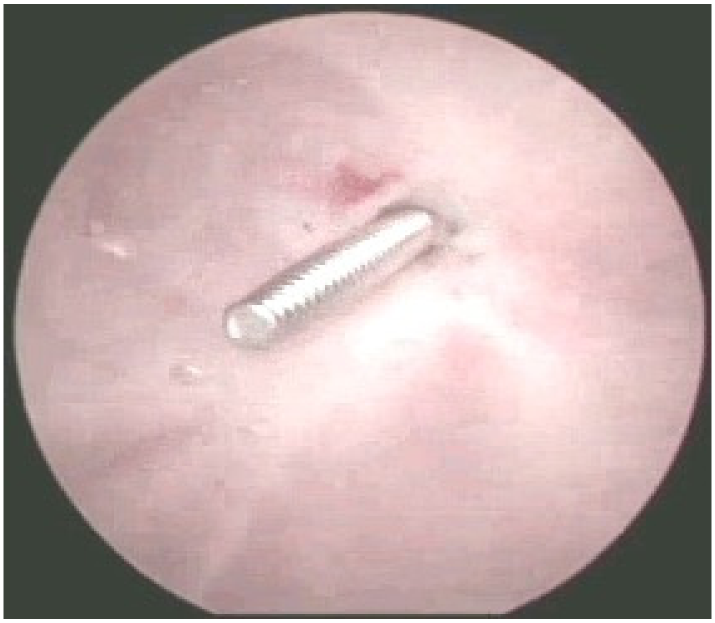

**Figure S2**. Tight (2mm) benign postcaustic stenosis - very hard scar
Patient with tight esophageal scar stenosis, with marked dysphagia after failed dilation attempts, with formal recommendation for esophagectomy and translational or visceral translocation, benefited from

successive, dimensionally progressive esophageal stenting (usually 3 stages), with brace directed scarring (over time).

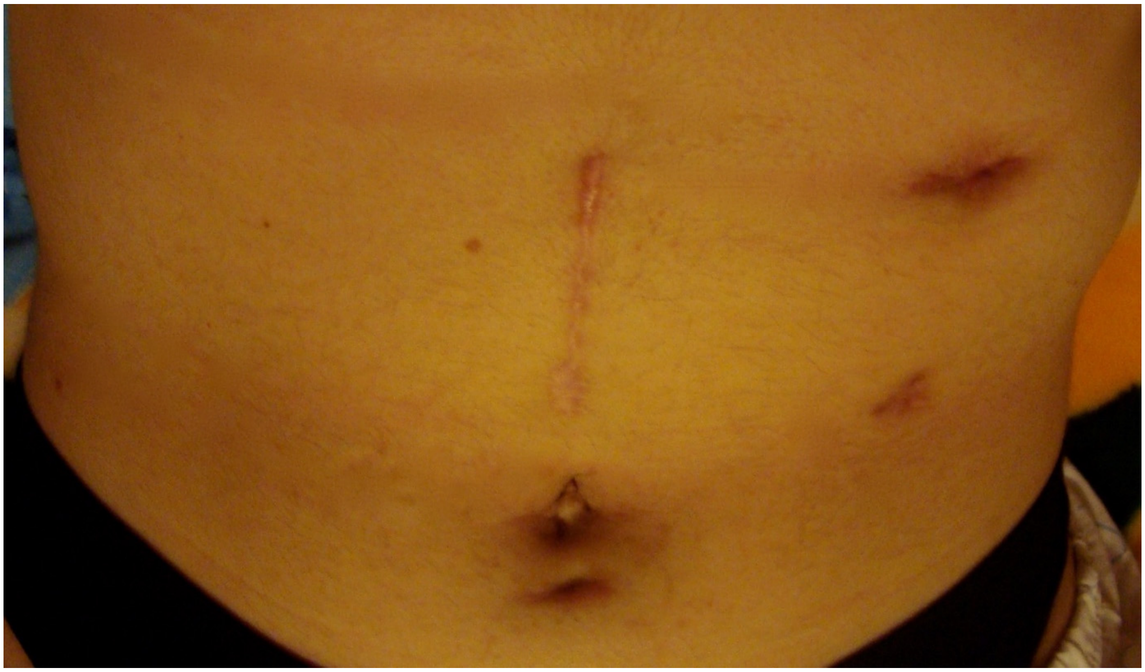

**Figure S3.** Postoperative image of patient with benign postcaustic stenosis and ruptured feeding jejunostomy. Patients with achalasia, liver cirrhosis, intra- and periesophageal portocaval anastomoses was stented (with stenosis healing on the stent).
